# 套细胞淋巴瘤患者维持治疗方案的多中心回顾性研究

**DOI:** 10.3760/cma.j.cn121090-20240118-00032

**Published:** 2024-07

**Authors:** 萍 杨, 澜 罗, 硕子 刘, 春源 李, 颖彤 陈, 薇 张, 辉 刘, 秀斌 肖, 红梅 景

**Affiliations:** 1 北京大学第三医院，北京 100191 Peking University Third Hospital, Beijing 100191, China; 2 北京协和医院，北京 100730 Peking Union Medical College Hospital, Beijing 100730, China; 3 北京医院，北京 100730 Beijing Hospital, Beijing 100730, China; 4 解放军总医院第五医学中心，北京 100039 The 5th Medical Center of PLA General Hospital, Beijing 100039, China

**Keywords:** 淋巴瘤，套细胞, 真实世界研究, 维持治疗, Lymphoma, mantle cell, Real-world study, Maintenance treatment strategies

## Abstract

**目的:**

探索套细胞淋巴瘤（MCL）患者不同维持治疗策略的生存差异。

**方法:**

收集1999年4月至2019年12月多家中心诊治的693例MCL患者临床资料，其中维持治疗组309例，总结不同维持治疗组患者的特征，进行Kaplan-Meier生存及预后分析。

**结果:**

本组研究总体3年和5年无进展生存（PFS）率分别为（73.5±2.9）％和（53.6±4.3）％，3年和5年总生存（OS）率分别为（94.2±1.5）％和（82.7±3.2）％。不同维持治疗策略组患者临床特征基本一致，利妥昔单抗组、来那度胺组、布鲁顿酪氨酸激酶抑制剂（BTKi）组、双药治疗组，3年PFS率分别为（70.4±4.1）％、（69.1±7.6）％、（86.9±5.0）％和（80.4±5.1）％，3年OS率分别为（92.9±2.4）％、（97.3±2.7）％、（97.9±2.1）％和（95.3±2.7）％，差异无统计学意义（*P*＝0.632、0.313）。多因素生存分析显示MCL国际预后指数（MIPI）高危组、维持治疗前完全缓解是PFS的独立危险因素；MIPI高危组、高剂量阿糖胞苷、治疗线数、早期疾病进展（POD24）为OS独立危险因素。

**结论:**

通过比较MCL的不同维持治疗策略，初步显示BTKi在维持治疗中有获益趋势，同时患者危险分层及维持治疗前缓解状态是影响疾病进展的重要因素。

## Abstract (en)

**Objective:**

This study aims to explore the survival advantages of different maintenance strategies for MCL.

**Methods:**

Clinical data of 693 newly diagnosed MCL patients in multi-centers admitted from April 1999 to December 2019 were collected. 309 cases received maintenance treatment. The characteristics of patients in different maintenance treatment groups were summarized and Kaplan-Meier survival and prognosis analysis were conducted.

**Results:**

The overall 3-year and 5-year progression-free survival（PFS）rates were（73.5±2.9）％ and（53.6±4.3）％, respectively. The 3-year and 5-year overall survival（OS）rates were（94.2±1.5）％ and（82.7±3.2）％, respectively. The clinical features of different maintenance treatment groups were generally consistent. The 3-year PFS rates of rituximab maintenance, lenalidomide maintenance, BTK inhibitor maintenance and dual-drug maintenance were（70.4±4.1）％,（69.1±7.6）％,（86.9±5.0）％, and（80.4±5.1）％, respectively. Corresponding 3-year OS rates were（92.9±2.4）％,（97.3±2.7）％,（97.9±2.1）％, and（95.3±2.7）％, respectively. There were no significant difference in different groups（*P*＝0.632, 0.313）. Survival analysis identified the MCL International Prognostic Index（MIPI）high-risk group and achieving complete remission before maintenance treatment as independent risk factors for PFS. The MIPI high-risk group, high-dose cytarabine application, treatment lines, and early disease progression（POD24）emerged as independent risk factors for OS.

**Conclusion:**

Comparing the different maintenance strategies of MCL, the result showed that BTK inhibitors（BTKi）maintenance demonstrated preliminary advantages in survival. Meanwhile, high-risk group according to MIPI and incomplete remission before maintenance treatment were significant factors related to disease progression.

套细胞淋巴瘤（MCL）是一种异质性较强的B细胞淋巴瘤，具有独特的组织学、免疫表型及细胞遗传学特征，兼具侵袭性淋巴瘤的侵袭性和惰性淋巴瘤不可治愈的特点，在西方国家占非霍奇金淋巴瘤的3％～10％，中位发病年龄为68岁[1]–[6]。50％～70％患者治疗缓解后会出现疾病复发或进展，尽管新药新方法如布鲁顿酪氨酸激酶抑制剂（BTKi）、Bcl-2抑制剂、来那度胺、靶向CD19嵌合抗原受体T（CD19 CAR-T）细胞免疫疗法和双特异性抗体等用于MCL的治疗并改善患者预后，但仍有部分患者出现耐药进展，尤其是早期复发患者，成为MCL治疗的难点，如何减少疾病复发是MCL治疗策略的重要组成部分[7]–[11]。

维持治疗是指在诱导治疗或巩固治疗后继续给予的治疗策略，可以进一步改善疾病控制、延长反应持续时间和减少疾病复发[12]。指南建议MCL患者在诱导治疗后使用利妥昔单抗作为维持治疗，亚洲淋巴瘤研究组共识建议亚洲MCL患者的维持方案可根据负担能力和报销情况进行个性化选择[13]。真实世界中，包括利妥昔单抗、BTKi和来那度胺等单药或联合治疗策略被用于MCL患者维持治疗[14]–[17]，不同维持治疗方案的疗效和生存优势，目前国内尚无报道。本研究通过多中心回顾性分析，收集不同维持治疗组临床资料，旨在进行疗效和生存分析，为临床治疗选择提供更多的依据。

## 病例与方法

1. 病例：本研究回顾性分析1999年4月至2019年12月在北京大学第三医院、中山大学肿瘤防治中心、解放军总医院第五医学中心、北京协和医院、中国医科大学附属第一医院、中日友好医院、北京同仁医院、北京医院、北京清华长庚医院、大连医科大学附属第二医院、哈尔滨医科大学附属第一医院、辽宁省肿瘤医院、吉林省肿瘤医院、中国医科大学盛京医院、哈尔滨医科大学附属肿瘤医院、包头市肿瘤医院、北京大学第一附属医院等共19家综合医院诊治的具有完整临床资料的693例MCL患者，其中309例在诱导治疗或巩固治疗结束后接受维持治疗。所有病例均经过病理组织活检和（或）免疫组织化学染色证实其诊断，且符合2008年WHO淋巴瘤分类诊断标准。

2. 维持治疗方案：309例患者进行维持治疗，其中152例患者接受利妥昔单抗维持治疗；43例接受来那度胺维持治疗，剂量10～25 mg/d，10～21 d，28 d为1个周期；47例患者接受BTKi维持治疗，伊布替尼560 mg/d或泽布替尼160 mg每天2次或奥布替尼150 mg/d，持续口服；67例患者接受R2（利妥昔单抗+来那度胺）方案或R-BTKi双药维持治疗。所有患者中位维持治疗时间为18.9（2～47）个月。

3. 分期及疗效评价：依照Ann Arbor分期标准进行临床分期，应用MCL国际预后指数（MIPI）和结合Ki-67的MCL国际预后指数（MIPI-c）对患者进行危险分层。疗效评价依据国际工作组（IWC）标准[4]，分为完全缓解（CR）、部分缓解（PR）、疾病稳定（SD）、疾病进展（PD）。

4. 生存评价：电话随访截至2022年1月，中位随访时间为49（4～168）个月。总生存（OS）期为从诊断之日起至任何原因导致死亡或随访终点的间隔时间。无进展生存（PFS）期为从诊断之日起至首次发现肿瘤进展、患者死亡或随访终点的间隔时间。所有患者均进行疗效及生存的评估。

5. 统计学处理：计数资料使用例（％）描述，计量资料使用*M*（范围）或*x±s*描述。应用SPSS 23.0统计软件进行统计学分析。多组之间分类变量数据比较采用卡方检验或Fisher精确检验；预后影响因素的单因素分析采用Kaplan-Meier和Log-rank检验，并绘制生存曲线；多因素生存分析采用Cox比例回归模型，*P*<0.05为差异具有统计学意义。

## 结果

1. 临床特征：309例维持治疗的MCL患者，男女比例为3.23∶1，中位年龄为58.6（31～88）岁，其中年龄≥65岁有89例（28.8％）。病理分型：白血病样非结节型13例（4.2％）；经典型255例（82.5％）；母细胞型/多形性41例（13.3％）。309例患者中Ann Arbor分期为Ⅲ/Ⅳ期279例（90.3％），初诊时伴有B症状92例（29.8％）、骨髓受累138例（44.7％）、胃肠道受累79例（25.6％）。MIPI中/高危组患者约占46.0％，MIPI-c高中危/高危组患者约占33.3％。一线治疗方面，116例（37.5％）应用高剂量阿糖胞苷的治疗方案，75例（24.3％）应用包含BTKi的治疗方案。48例（15.5％）患者接受auto-HSCT作为巩固治疗；维持治疗前疗效评估，CR 177例（57.3％）、PR 113例（36.6％）、治疗无效19例（6.2％）。

本研究进一步比较不同维持方案组患者基本临床特征，见表1。在年龄、分期、B症状、病理分型、Ki-67≥50％、骨髓受累、胃肠道受累、MIPI中高危组及MIPI-c高中危/高危组比例等临床特征方面，三组组间差异无统计学意义；在一线治疗方面，利妥昔单抗组患者应用高剂量阿糖胞苷比例更高；而BTKi组及双药维持治疗组中应用BTKi联合治疗比例更高，auto-HSCT在各组之间差异无统计学意义。维持治疗前疾病状态，利妥昔单抗及来那度胺组CR患者比例高于BTKi组及双药维持治疗组（65.1％、65.1％对44.7％、43.3％，*P*＝0.008）。

**表1:** 套细胞淋巴瘤患者不同维持治疗组的临床特征［例（％）］

临床特征	利妥昔单抗组 （152例）	来那度胺组 （43例）	BTKi组 （47例）	双药维持治疗组 （67例）	*P*值
年龄≥65岁	39（25.7）	12（27.9）	17（36.2）	21（31.3）	0.531
Ann Arbor Ⅲ～Ⅳ期	136（89.5）	39（90.7）	45（95.7）	59（88.1）	0.552
病理分型					0.220
经典型	128（84.2）	37（86.0）	37（78.7）	53（79.1）	
母细胞/多形性	22（14.5）	3（7.0）	6（12.8）	10（14.9）	
B症状	45（29.6）	12（27.9）	14（29.8）	21（31.3）	0.985
Ki-67≥50%	37（24.3）	9（20.9）	13（27.7）	15（22.4）	0.880
骨髓受累	68（44.7）	17（39.5）	22（46.8）	37（46.2）	0.893
胃肠道受累	41（26.9）	9（20.9）	13（27.7）	16（23.9）	0.837
MIPI:中/高危组	69（44.7）	17（39.5）	25（53.2）	31（46.3）	0.491
MIPI-c:高中危/高危组	49（32.2）	10（23.3）	20（42.6）	24（35.8）	0.444
一线治疗					
高剂量阿糖胞苷治疗	68（44.7）	13（30.2）	13（27.7）	22（32.8）	0.074
BTKi	15（9.9）	2（4.7）	28（59.6）	30（44.8）	<0.001
auto-HSCT	23（15.1）	9（20.9）	6（12.8）	10（14.9）	0.737
维持治疗前缓解状态					0.008
完全缓解	99（65.1）	28（65.1）	21（44.7）	29（43.3）	
部分缓解	50（32.9）	13（30.2）	21（44.7）	29（43.3）	

**注** MIPI：套细胞淋巴瘤国际预后指数；MIPI-c：结合Ki-67的套细胞淋巴瘤国际预后指数；BTKi：布鲁顿酪氨酸激酶抑制剂

2. 维持治疗生存分析：中位随访（49±2.0）个月，患者总体的中位PFS期为（61.0±4.3）个月，中位OS期为（151.0±47.7）个月。3年及5年PFS率分别为（73.5±2.9）％和（53.6±4.3）％，3年及5年OS率分别为（94.2±1.5）％和（82.7±3.2）％。

不同维持方案比较，利妥昔单抗组患者3年PFS率为（70.4±4.1）％，来那度胺组为（69.1±7.6）％，BTKi组为（86.9±5.0）％，双药维持治疗组为（80.4±5.1）％，各组间比较差异无统计学意义（*P*＝0.632）；利妥昔单抗组患者3年OS率为（92.9±2.4）％，来那度胺组为（97.3±2.7）％，BTKi组为（97.9±2.1）％，双药维持治疗组为（95.3±2.7）％，各组间比较差异无统计学意义（*P*＝0.313）。

3. 预后因素分析：单因素分析显示年龄≥65岁、MIPI中/高危组、MIPI-c高中危/高危组、诱导治疗中未使用高剂量阿糖胞苷、未行auto-HSCT作为巩固治疗、维持治疗前未达CR为本组研究PFS的预后不良因素（*P*值均<0.05）；年龄≥65岁、MIPI中/高危组、MIPI-c高中危/高危组、诱导治疗中未使用高剂量阿糖胞苷、维持治疗前未达CR、Ki-67≥50％、存在结外器官累及、治疗线数≥3线、24个月内疾病进展（POD24）为本组研究OS的预后不良因素（*P*值均<0.05）。Cox比例回归模型多因素分析显示MIPI中/高危组、维持治疗前未达CR是MCL维持治疗组患者PFS的独立危险因素；MIPI中/高危组、诱导治疗中未使用高剂量阿糖胞苷、治疗线数≥3线、POD24为OS独立危险因素（表2）。

**表2:** 309例接受维持治疗的套细胞淋巴瘤患者的多因素分析

	纳入指标	*HR*（95% *CI*）	*P*值
无进展生存	MIPI中/高危组	1.548（1.187～2.017）	0.001
	维持治疗前未达完全缓解	2.532（1.716～3.738）	<0.001
总生存	MIPI中/高危组	2.545（1.603～4.042）	<0.001
	未使用高剂量阿糖胞苷	0.365（0.140～0.950)	0.039
	治疗线数≥3线	1.410（1.093～1.819）	0.008
	24个月内疾病进展	2.334（1.651～3.300）	<0.001

**注** MIPI：套细胞淋巴瘤国际预后指数

## 讨论

由于基因组学的不稳定性，50％～70％的MCL患者会出现疾病复发或难治，一旦复发，尽管存在各种靶向新药、CAR-T细胞治疗及双特异性抗体等治疗手段，但总体预后差。维持治疗是延缓和减少MCL复发的重要治疗手段，是目前指南推荐的治疗策略。维持治疗药物中，除利妥昔单抗外，靶向新药的维持治疗策略在临床研究中显示具有生存的获益。在真实世界中MCL患者维持治疗能否有更好的获益，尚缺乏临床研究数据。

意大利淋巴瘤基金会（FIL）多中心前瞻对照研究[16]显示初治的MCL患者在R-CHOP/高剂量阿糖胞苷治疗获得CR或PR后进行auto-HSCT作为巩固治疗，采用来那度胺维持治疗，结果显示中位随访38个月，来那度胺组较对照组PFS期延长（3年PFS率：80％对64％，*P*＝0.012）；39％患者因死亡或疾病进展停用来那度胺治疗。与对照组相比，来那度胺组3～4级血液学毒性及感染等不良反应显著增加。硼替佐米作为蛋白酶体抑制剂，既往研究[18]显示VR-CAP（硼替佐米+利妥昔单抗+环磷酰胺+阿霉素+泼尼松）方案在MCL具有较好的疗效，其作为维持治疗策略，目前仅有小样本研究。HOVON75研究[19]在初治MCL患者R-CHOP/高剂量阿糖胞苷联合auto-HSCT后应用硼替佐米1.3 mg/m^2^进行维持治疗，结果显示4年无事件生存率及OS率较对照组差异无统计学意义。SWOGS0601研究[20]显示初治MCL应用VR-CHOP方案6个周期后予硼替佐米维持治疗2年，结果显示维持治疗过程中最佳CR率为57％，总缓解率为83％，2年PFS率为62％、2年OS率为85％，高于既往未使用硼替佐米的部分研究，对于硼替佐米作为维持治疗具体剂量及获益仍需进一步研究证实。

BTKi作为MCL的里程碑式药物，在初治及复发难治MCL中有广泛应用，对于BTKi维持治疗也有临床研究探索。Karmali等[17]研究显示初始治疗联合auto-HSCT获得CR或PR后，进行伊布替尼单药维持治疗4年，同时进行基于二代测序的微小残留病监测，研究显示伊布替尼维持组5年PFS率和OS率分别为89％和91％；最常见不良反应为感染（86％），3～4级不良反应为血液学毒性，42％因药物不良反应导致维持治疗停止，其中最常见原因为新发房颤/房扑。SHINE研究[21]中对于老年初治MCL患者采用BR（苯达莫司汀和利妥昔单抗）联合BTKi治疗后，应用BTKi联合利妥昔单抗维持治疗，结果显示BTKi维持治疗组相对于对照组有显著PFS生存获益（中位PFS期80.6个月对52.9个月，*P*＝0.023），OS差异无统计学意义；但3级或4级不良反应发生率为81.5％，房颤占13.9％，肺炎占20.1％。因此，BTKi维持治疗中仍需高度关注不良反应，尤其是老年患者。

与既往研究[22]相似，本研究显示维持治疗组患者在PFS及OS均具有优势。进一步对不同维持治疗方案进行分析，结果显示BTKi维持治疗组在3年PFS及OS率高于其他维持治疗方式，这可能与研究中使用新型BTKi减少药物不良反应从而保证维持治疗能够持续进行相关。同时，双药维持治疗组（R2/R-BTKi）较单药治疗未显示明显生存优势，可能与双药维持治疗组随访时间不足有关。与利妥昔单抗组比较，BTKi及双药维持治疗组，在维持治疗前CR比例低（44.7％、43.3％对65.1％），但PFS及OS率更高，原因可能与上述两组患者初始治疗使用BTKi比例明显升高相关。上述研究显示BTKi在维持治疗优势，但仍需后继进一步扩大样本量、延长随访时间及开展随机对照研究，进一步验证双药维持治疗及BTKi维持治疗能否作为最佳维持治疗方案。

维持治疗组进一步生存分析显示MIPI中/高危组、诱导治疗后未达CR是影响PFS的独立危险因素。Karmali等[17]研究显示伊布替尼维持治疗前接受auto-HSCT患者具有更高的5年PFS率（100％对77％）和OS率（100％对83％）；欧洲MCL协作组的TRIANGLE研究同样显示在年轻适合移植的患者中应用BTKi维持治疗具有更好的生存获益[23]。本研究同样显示在适合移植的年轻患者中维持治疗有更进一步生存获益，同时维持治疗前缓解状态是患者能否获得长期生存重要因素，维持治疗前获得CR能够有更好的PFS，而PR或SD患者在维持治疗后更易发生PD。

综上所述，本研究对真实世界中MCL患者维持治疗方案进行探讨，全面描述了维持治疗组患者临床特征，对不同维持治疗方案进行生存及预后分析，通过大样本真实世界数据验证MCL维持治疗的生存优势，在不同维持治疗方案中，BTKi初步显示其在生存方面获益，同时维持治疗前危险分层及缓解状态是影响维持治疗组患者疾病进展的重要因素。本研究为回顾性分析，随访时间尚短，需要进一步延长随访时间及随机对照验证最佳维持治疗方案。
